# Crystal structures of *Aspergillus oryzae* Rib2 deaminase: the functional mechanism involved in riboflavin biosynthesis

**DOI:** 10.1107/S205225252100275X

**Published:** 2021-05-05

**Authors:** Sheng-Chia Chen, Li-Ci Ye, Te-Ming Yen, Ruei-Xin Zhu, Cheng-Yu Li, San-Chi Chang, Shwu-Huey Liaw, Chun-Hua Hsu

**Affiliations:** a Department of Life Sciences and Institute of Genome Sciences, National Yang-Ming University, Taipei 11221, Taiwan; b Department of Agricultural Chemistry, National Taiwan University, Taipei 10617, Taiwan; c Institute of Biochemistry and Molecular Biology, National Yang-Ming University, Taipei 11217, Taiwan; d Department of Chemistry, National Taiwan University, Taipei 10617, Taiwan; e Department of Medical Research and Education, Taipei Veterans General Hospital, Taipei 11217, Taiwan; f Institute of Biochemical Sciences, National Taiwan University, Taipei 10617, Taiwan; g Genome and Systems Biology Degree Program, National Taiwan University and Academia Sinica, Taipei 10617, Taiwan

**Keywords:** riboflavin biosynthesis, Rib2, deaminases, substrate binding, crystal structure

## Abstract

Riboflavin serves as the direct precursor of the FAD/FMN coenzymes and is biosynthesized in most prokaryotes, fungi and plants. Fungal Rib2 possesses a deaminase domain for deamination of pyrimidine in the third step of riboflavin biosynthesis. Here, four high-resolution crystal structures of a Rib2 deaminase from *Aspergillus oryzae* (AoRib2) are reported which display three distinct occluded, open and complex forms that are involved in substrate binding and catalysis. In addition to the deaminase domain, AoRib2 contains a unique C-terminal segment which is rich in charged residues. Deletion of this unique segment has no effect on either enzyme activity or protein stability. Nevertheless, the C-terminal αF helix preceding the segment plays a role in maintaining protein stability and activity. Unexpectedly, AoRib2 is the first mononucleotide deaminase found to exist as a monomer, perhaps due to the assistance of its unique longer loops (L_β1–β2_, L_αB–β3_ and L_αC–β4_). These results form the basis for a molecular understanding of riboflavin biosynthesis in fungi and might assist in the development of antibiotics.

## Abstract 2

Crystal structures of Rib2 deaminase reveal its substrate specificity and catalytic mechanism.

## 1. Introduction   

The FAD/FMN coenzymes derived from riboflavin are essential for a wide variety of physiological processes and hence occur in all organisms (Macheroux *et al.*, 2011 ▸). Their precursor riboflavin is biosynthesized in most prokaryotes, fungi and plants (García-Angulo, 2017 ▸; Haase *et al.*, 2014 ▸; Roje, 2007 ▸). In contrast, animals lack this biosynthetic pathway and hence must obtain this vitamin from nutritional sources. Some pathogenic microorganisms are unable to take up riboflavin from the environment and can only obtain this vitamin by biosynthesis. Therefore, the enzymes of riboflavin biosynthesis have potential as antimicrobial drug targets, particularly for the development of new chemotherapeutic agents against antibiotic-resistant pathogens (Haase *et al.*, 2014 ▸; Kaiser *et al.*, 2007 ▸; Long *et al.*, 2010 ▸; Meir & Osherov, 2018 ▸).

**Figure 1:**
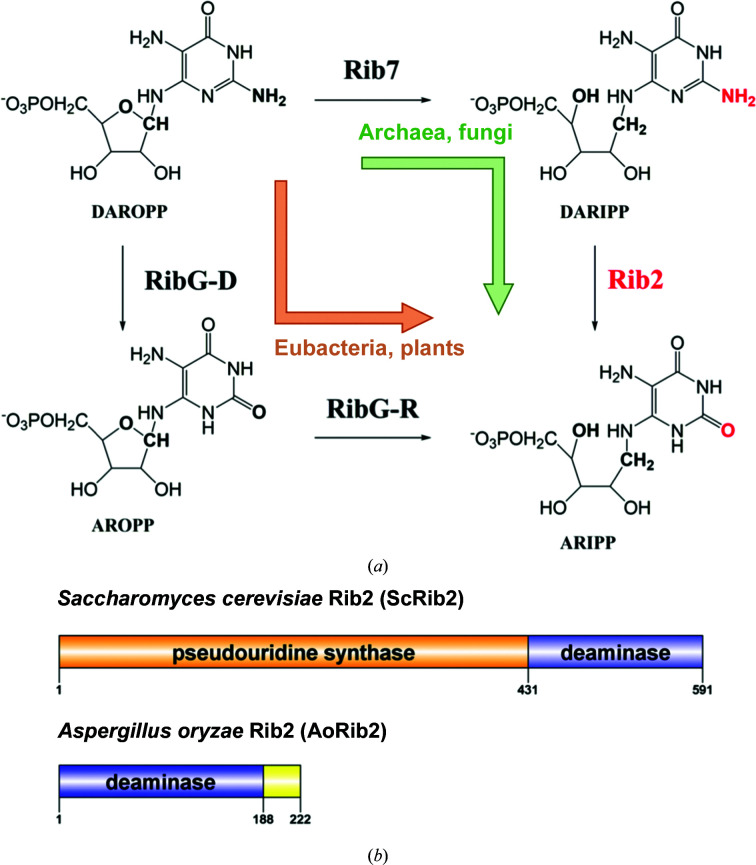
(*a*) The deamination and reduction steps in vitamin B_2_ biosynthesis. (*b*) Domain compositions of ScRib2 and AoRib2. The pseudouridine synthase domain is shown in orange and the deaminase domains are shown in blue. A charged residue-rich segment is shown in yellow.

In the riboflavin-biosynthesis pathway, GTP cyclohydrolase II first catalyzes the conversion of GTP to 2,5-diamino-6-ribosyl­amino-4(3*H*)-pyrimidinone 5′-phosphate (DAROPP; Richter *et al.*, 1993 ▸). In eubacteria and plants, DAROPP is deaminated to 5-diamino-6-ribosyl­amino-2,4(1*H*,3*H*)-pyrimidinedione 5′-phosphate (AROPP) and is then reduced to 5-amino-6-ribityl­amino-2,4(1*H*,3*H*)-pyrimidinedione 5′-phosphate (ARIPP) [Fig. 1 ▸(*a*)]. In most eubacteria, the enzyme responsible is the bifunctional protein RibG (or RibD), which comprises an N-terminal deaminase domain and a C-terminal reductase domain (Richter *et al.*, 1997 ▸). Recently, we solved the tetrameric structures of *Bacillus subtilis* RibG (BsRibG) in complex with the NADPH cofactor and substrate (Chen *et al.*, 2006 ▸, 2009 ▸, 2013 ▸). The reductase domain and dihydrofolate reductase, a pharmaceutically important enzyme, share a conserved core structure containing the NADPH-binding site and possess a similar catalytic mechanism (Chen *et al.*, 2009 ▸). The deaminase domain belongs to the cytidine deaminase (CDA) superfamily, which includes the mononucleotide deaminases involved in nucleotide metabolism and the RNA/DNA-editing deaminases associated with gene diversity and antivirus defense (Knisbacher *et al.*, 2016 ▸; Knisbacher & Levanon, 2015 ▸).

Intriguingly, the biosynthesis pathway for riboflavin in archaea and fungi is slightly different from that in plants and eubacteria. In this pathway, DAROPP is reduced to 2,5-diamino-6-ribityl­amino-4(3*H*)-pyrimidinone 5′-phosphate (DARIPP) by Rib7 and subsequently deaminated to ARIPP by Rib2 [Fig. 1 ▸(*a*)] (Graupner *et al.*, 2002 ▸; Behm-Ansmant *et al.*, 2004 ▸). Therefore, the deaminase domains of RibG and Rib2 possess distinct substrate specificities. The deaminase domain of fungal Rib2 enzymes also belongs to the CDA superfamily and contains conserved H*X*E and PC*XX*C signatures; nevertheless, these enzymes vary widely in length and show obvious distinct variations in sequence and in the structural domains that are present. For example, yeast Rib2 usually comprises an N-terminal pseudouridine synthase domain and a C-terminal deaminase domain (Behm-Ansmant *et al.*, 2004 ▸). However, mold Rib2 only contains the deaminase domain [Fig. 1 ▸(*b*)]. In other words, only the deaminase domain is involved in the riboflavin-biosynthesis pathway.

To investigate the deaminase in the biosynthetic pathway specific to archaea and fungi, we solved the crystal structure of *Methanosarcina mazei* Rib7 (MmRib7; Chen *et al.*, 2018 ▸). Because structural information regarding fungal Rib2 remains unavailable, a three-dimensional structural study of Rib2 would further expand our understanding of the structural differences between the fungal and eubacterial pyrimidine deaminases involved in riboflavin biosynthesis. Nevertheless, all attempts to obtain crystal structures of the Rib2s from *Pichia pastoris* and *Saccharomyces cerevisiae* (ScRib2) have been unsuccessful. As an alternative, a 222-residue putative Rib2 deaminase from *Aspergillus oryzae* was chosen for improved characterization. Like ScRib2 (Behm-Ansmant *et al.*, 2004 ▸), the recombinant protein is able to catalyze the conversion of DARIPP to ARIPP and hence is named AoRib2. To gain insight into the substrate specificity and catalytic mechanism of a fungal pyrimidine deaminase involved in riboflavin biosynthesis, we performed crystallo­graphic and biochemical characterization of AoRib2. We have determined the X-ray structures of the open and occluded forms, as well as of the complex with the substrate DARIPP. Our results provide a detailed picture of the structure–function relationship in Rib2 and shed light on the roles of the active-site residues involved in ligand binding and catalysis.

## 2. Methods   

### 2.1. Cloning, expression and purification of AoRib2   

AoRib2 was expressed using the pET-21b vector in *Escherichia coli* Rosetta (DE3) cells. The recombinant protein contains eight additional vector residues (LEHHHHHH) at the C-terminus. The cell pellets were resuspended in lysis buffer consisting of 20 m*M* sodium phosphate pH 7.5, 500 m*M* NaCl, 1 m*M* phenylmethanesulfonyl fluoride and lysed by sonication. After the removal of cellular debris by centrifugation at 20 000*g* at 277 K for 30 min, the crude extract was applied onto a 6 ml nickel–nitrilotriacetic acid column. After washing with 20, 40 and 60 m*M* imidazole, the protein was eluted with 250 m*M* imidazole. Fractions containing AoRib2 were dialysed and then pooled for additional purification using a Q Sepharose column (GE Healthcare). The protein eluted at 10 m*M* Tris–HCl pH 7.5, 100 m*M* NaCl, 1 m*M* dithiothreitol. AoRib2 was pooled and concentrated to 14 mg ml^−1^ for crystallization screening. In addition, AoRib2_188_ and AoRib2_177_ mutations were introduced into pET-21b. Expression and purification of the deletion mutants were performed as described above.

### 2.2. Crystallization and data collection   

The initial crystallization screening was performed with Hampton Research Crystal Screens using the hanging-drop vapor-diffusion method at 288 K. Crystals of AoRib2 and AoRib2_188_ were grown using a combination of 2 µl reservoir solution and 2 µl protein solution (14 mg ml^−1^). Crystals appeared and reached final dimensions of ∼0.1 × 0.1 × 0.3 mm within 10–14 days at 288 K. X-ray diffraction data were collected at 110 K with the addition of 20% glycerol as a cryoprotectant. Data were collected at the National Synchrotron Radiation Research Center, Taiwan. An AoRib2 crystal was used for single-wavelength anomalous dispersion (Zn-SAD) data collection at the peak wavelength on the TPS-05A beamline (λ = 1.2824 Å and 50% beam attenuation). The DARIPP derivative was prepared by soaking crystals for 20 min in reservoir solution containing 50–100 m*M* DARIPP. Diffraction data were processed using *HKL*-2000 (Otwinowski & Minor, 1997 ▸). The resolution cutoff of our diffraction data was at an average *I*/σ(*I*) of >2 in the highest resolution shell. The data statistics are summarized in Table 1 ▸.

### 2.3. Structure determination   

The initial phase was obtained by the Zn-SAD method using *Phaser* (McCoy *et al.*, 2007 ▸) and an *ab initio* model was built using *ARP*/*wARP* (Carolan & Lamzin, 2014 ▸). One zinc site was identified and the initial phases calculated from the site were further improved by density modification. The resulting electron-density map was used to build most of the AoRib2 structure. The structures of the apo form and binary complex of AoRib2_188_ were solved using *Phaser* (McCoy *et al.*, 2007 ▸) with AoRib2 as the search model. The structures were completed via multiple manual interactions in *Coot* (Emsley *et al.*, 2010 ▸). Structural refinement was carried out using *REFMAC* (Murshudov *et al.*, 2011 ▸) and *Phenix* (Liebschner *et al.*, 2019 ▸). The figures showing protein structures were prepared with *PyMOL* (http://www.pymol.org/).

### 2.4. Biophysical characterization   

The molecular mass in solution was estimated using a Beckman–Coulter XL-A Analytical Ultracentrifuge with an An60Ti rotor. Prior to the experiments, the samples were diluted to a protein concentration of 0.3 mg ml^−1^ with 20 m*M* phosphate-buffered saline (PBS) pH 7.5. Sedimentation-velocity analysis was performed at 293 K and 42 000 rev min^−1^ with standard double-sector centerpieces. The UV absorption of the cells was scanned every 5 min for 6 h and the data were analyzed using *SEDFIT* (Schuck, 2000 ▸).

CD spectra were recorded at 5°C and a protein concentration of 0.5 mg ml^−1^ in 20 m*M* sodium phosphate pH 7.5 with a cuvette of 0.1 cm path length. Thermal unfolding profiles between 25 and 95°C were recorded by increasing the temperature at a ramping rate of 1°C min^−1^ while monitoring the CD signal at 222 nm. The midpoints of thermal unfolding were calculated using *SigmaPlot*.

### 2.5. Deaminase activity assay   

DAROPP was prepared by the addition of 0.5 *M* GTP to a solution of *E. coli* GTP cyclohydrolase II (5 mg ml^−1^) to a final concentration of 10–30 m*M*. The reaction was complete in about 10–15 min; *P. pastoris* Rib7 (1 mg) and 25 m*M* NADPH were added for 20 min to produce DARIPP. The activities of wild-type and mutant AoRib2 were assayed using the changes in fluorescence intensity after diacetyl modification. The reaction mixtures were incubated for 30 min at 310 K. Diacetyl was added to a final concentration of 1%(*v*/*v*) and the mixture was incubated at 368 K for 10 min. The fluorescence spectra were recorded using a Perkin Elmer LS-50B luminescence spectrometer.

## 3. Results   

### 3.1. Overall structure of AoRib2   

**Figure 2:**
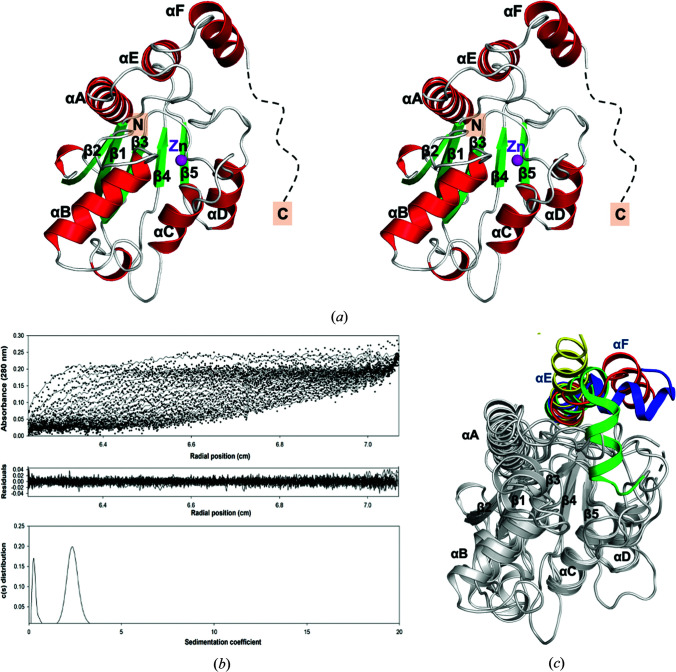
Structure of AoRib2. (*a*) The deaminase domain is made up of a central β-sheet flanked by α-­helices. The endogenous zinc ion in the deaminase domain is shown as a magenta sphere. (*b*) Sedimentation-velocity analysis of AoRib2. The enzyme concentration was 0.3 mg ml^−1^ in 20 m*M* PBS pH 7.5. The three panels represent the optical traces (top), the residuals of the model fitting (middle) and the sedimentation-coefficient distribution (bottom). The ultracentrifugation analysis demonstrated that AoRib2 exists as a monomer in solution. (*c*) Structural superposition of AoRib2 (red), yCD (green), BsRibG (blue) and EcTadA (yellow). They share a similar structural core (gray) but show divergence in the C-terminal helices.

AoRib2 is composed of a deaminase domain (residues 1–188) and a flexible C-terminal region (residues 189–222) rich in charged residues. Both the full length and the deaminase domain (AoRib2_188_) of AoRib2 were bacterially produced and crystallized. AoRib2 crystals in space group *P*4_1_2_1_2 were grown in 1.5 *M* ammonium sulfate, 0.1 *M* sodium acetate pH 4.6 (AoRib2^pH4.6^), while crystals of AoRib2_188_ were grown in two different space groups. Crystals of AoRib2_188_ with space group *P*4_1_2_1_2 were obtained by equilibration against a reservoir solution consisting of 2 *M* sodium chloride, 0.1 *M* sodium acetate pH 4.6 (AoRib2_188_^pH4.6^) and crystals of AoRib2_188_ with space group *P*2_1_2_1_2 were obtained using a reservoir solution consisting of 0.1 *M* MES pH 6.5, 0.2 *M* ammonium sulfate, 30% PEG 5000 MME (AoRib2_188_^pH6.5^). There is one molecule in the asymmetric unit for all three crystals. Interestingly, the atomic model of AoRib2^pH4.6^ contains only residues 3–188 due to unclear electron density for the C-terminal region after residue 188, indicating relatively high thermal motion and a disordered conformation. The structure of the deaminase domain is composed of a central five-stranded β-sheet (β1–β5) sandwiched by three α-helices (αA, αE and αF) on one side and three α-helices (αB–αD) on the other side [Fig. 2 ▸(*a*)]. AoRib2 has a conserved core architecture akin to that of CDA superfamily members.

Compared with deaminases of known structure involved in riboflavin biosynthesis, which present an oligomeric form, analytical ultracentrifugation (AUC) experiments clearly demonstrated that AoRib2 exists as a monomer in solution [Fig. 2 ▸(*b*)]. A previous study reported that the N-terminal deaminase domain (residues 1–147) of BsRibG was sufficient for enzyme activity, while the truncated protein could not be purified because of poor stability (Richter *et al.*, 1997 ▸). Unlike the deaminase domain of BsRibG (BsRibG-D), the deamin­ase domain of AoRib2 could be purified and stabilized in solution. Comparing the molecular size, the deaminase domain of AoRib2 is larger than BsRibG-D. The additional residues of the AoRib2 deaminase domain are located in the L_β1–β2_, L_αB–β3_ and L_αC–β4_ loops. These three loops are ordered and participate in hydrogen bonds and hydrophobic inter­actions, which may make a significant contribution to stabilizing the core structure of AoRib2. Moreover, β-strands (β1 and β3) and helix αB of AoRib2 are also involved in the interaction of these three loops. The additional interactions lead to an increase in the number of hydrophobic patches in the core structure, which may result in the structural stability of AoRib2 being greater than that of the truncated BsRibG protein.

### 3.2. Structural comparison with other CDA deaminases   

Based on a structural alignment search using the *DALI* server, AoRib2 shares high structural similarity with other CDA superfamily members, including BsRibG (PDB entry 4g3m; *Z*-score 16.9; 25% identity; Chen *et al.*, 2013 ▸), yCD (PDB entry 1uaq; *Z*-score 14.5; 23% identity; Ko *et al.*, 2003 ▸) and EcTadA (PDB entry 1z3a; *Z*-score 14.0; 24% identity; Kim *et al.*, 2006 ▸). The β-sheet (β1–β5) and three α-helices (αA–αC) of AoRib2 are virtually superimposable on the structures of these CDA members, but the C-terminal segment differs significantly [Fig. 2 ▸(*c*)]. The C-terminal αF helix of yCD forms a cap narrowing the opening of the active-site cavity for the nucleobase, whereas the C-terminal helices in EcTadA, AoRib2 and BsRibG swing the active-site cavity away to accommodate their larger substrates (Kuratani *et al.*, 2005 ▸; Losey *et al.*, 2006 ▸). The C-terminal helix of EcTadA is involved in tRNA inter­action. Nonetheless, the C-terminal helices of AoRib2 and BsRibG do not participate in substrate binding, thus contributing to maintaining structural integrity.

### 3.3. DARIPP recognition by AoRib2   

**Figure 3:**
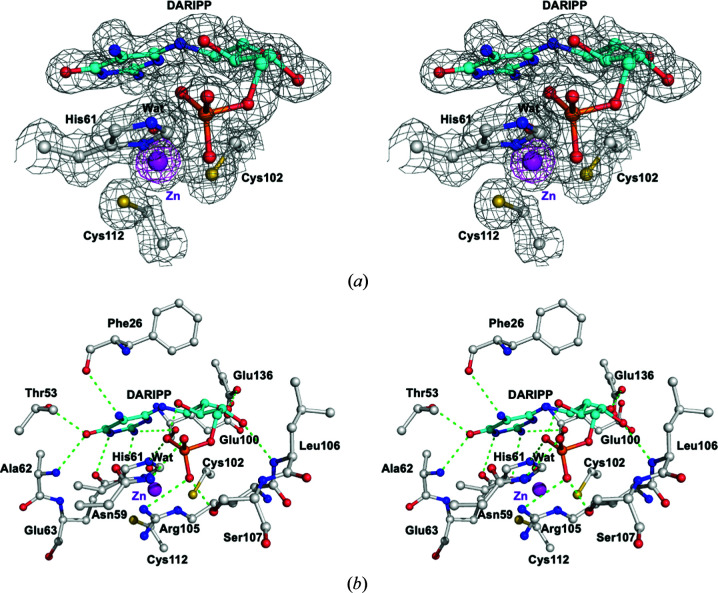
The DARIPP-binding site in AoRib2. (*a*) The 2*F*_o_ − *F*_c_ composite OMIT map of the active site contoured at the 1.0σ and 8.0σ levels and drawn as gray and magenta mesh presentations, respectively. (*b*) Interaction networks between DARIPP and AoRib2. DARIPP is displayed in cyan. Hydrogen bonds are shown as green dashed lines.

AoRib2 is a member of the CDA superfamily, with conserved H*X*E and PC*X*_8–9_C tetrahedral zinc-binding motifs (Chen *et al.*, 2006 ▸). The Zn^2+^ ion is coordinated by His61 N^δ1^ (2.0 Å), Cys102 S^γ^ (2.2 Å) and Cys112 S^γ^ (2.3 Å), and a fourth coordination is provided by a water molecule. The zinc-bound water molecule interacts with Glu63 O^ɛ2^ (2.6 Å). The zinc ion and Glu63 are proposed to activate the zinc-bound water required for the deamination activity (Betts *et al.*, 1994 ▸). In the absence of a substrate, the structure of ligand-free AoRib2 reveals that the active site of the enzyme is occupied by water and a sulfate ion, which occupies the phosphate-binding site. Hence, we reduced the concentration of ammonium sulfate and performed ligand-soaking experiments. As the result of ligand soaking, DARIPP could be built nicely into strong electron density and is firmly embedded in the active site of the AoRib2 crystal structure [Fig. 3 ▸(*a*)]. Analysis of the inter­action reveals that the pyrimidine N^3^H group of DARIPP interacts with the side-chain O^ɛ1^ atom of Glu63 (3.1 Å), the O^4^ atom of DARIPP interacts with the backbone N atom of Ala62 (2.9 Å) and the side-chain O^γ1^ atom of Thr53 (2.6 Å), the N^5^H_2_ group of DARIPP interacts with the backbone carbonyl group of Phe26 (3.2 Å), and the N^2^H_2_ group of DARIPP interacts with the side-chain O^ɛ2^ atom of Glu63 (3.2 Å) and the backbone carbonyl group of Glu100 (3.5 Å). In addition, the O^2′^H, O^3′^H and O^4′^H groups of the ribitol make close contacts with the side-chain O^ɛ2^ atom of Glu136 (2.7 Å), its phosphate group (2.6 Å) and the backbone N atom of Leu106 (3.0 Å), respectively. The phosphate moiety of DARIPP forms hydrogen bonds to the side-chain atoms N^δ2^ of Asn59 (3.0 Å), N^δ2^ of His61 (2.8 Å), N^η1^ of Arg105 (3.0 Å) and O^γ^ of Ser107 (2.6 Å) [Fig. 3 ▸(*b*)]. Of note, an intramolecular hydrogen bond is formed in the ligand between the O^3′^H group and the phosphate moiety. Moreover, the side chain of His61 stacks against the pyrimidine ring with an interplanar distance of about 3.5 Å. The substrate-interacting residues are all conserved in the fungal Rib2.

**Figure 4:**
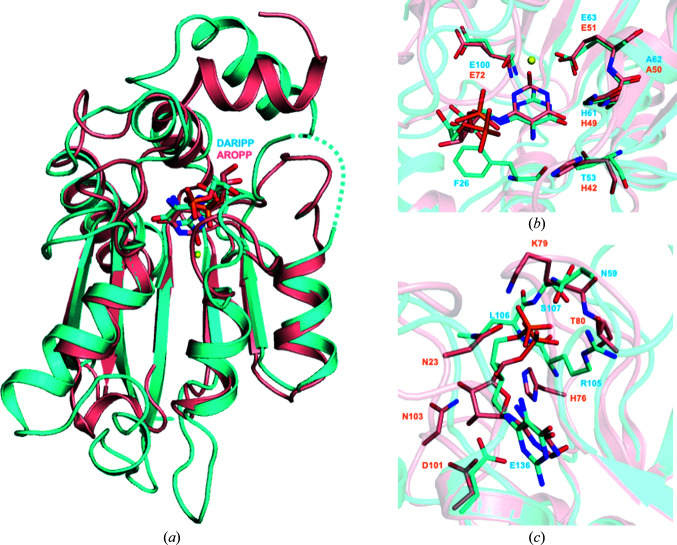
Structural comparison of AoRib2–DARIPP and BsRibG–AROPP. (*a*) Overall structure comparison of the deaminase domains of AoRib2 (cyan) and BsRibG (salmon). (*b*) Superposition of the structures of AoRib2–DARIPP and BsRibG–AROPP showing the evolutionary conservation of nucleobase-recognition residues. (*c*) Superposition of the structures of AoRib2–DARIPP and BsRibG–AROPP showing the structural divergence of phosphate- and ribose-recognition residues.

In eubacteria and fungi, the deamination and reduction steps in riboflavin biosynthesis proceed in the opposite order [Fig. 1 ▸(*a*)]. Therefore, the substrates of RibG and Rib2 are distinct: DAROPP with a cyclic ribose versus DARIPP with a linear ribitol. Structural superimposition of DARIPP-bound AoRib2 and AROPP-bound BsRibG-D revealed that the conformations of their active-site loops are quite different [Fig. 4 ▸(*a*)]. The residues of the two structures involved in the recognition of the pyrimidine moiety of DARIPP and AROPP occupy an equivalent spatial position [Fig. 4 ▸(*b*)], while the residues of AoRib2 involved in the recognition of the ribitol (or ribose for BsRibG) and phosphate moieties are distinct [Fig. 4 ▸(*c*)]. In addition, among the phosphate-interacting residues in AoRib2, Arg105 seems to be particularly important because it forms a salt bridge with the phosphate group of DARIPP. Lys79 in BsRibG plays a similar role to Arg105 in AoRib2 [Fig. 4 ▸(*c*)]. A notable structural difference involves the side chains of Asp101 and Asn103 in BsRibG that enable a direct interaction with O^2′^H and O^3′^H of the ribose, whereas Leu106 and Glu136 in AoRib2 make close contacts with O^4′^H and O^2′^H of ribitol.

The substrate-interacting loops of AoRib2 and BsRibG-D adopt a different conformation for ligand binding. In BsRibG, AROPP induces a significant conformation involving the L_β3–αC_ and L_β4–αD_ loops, which move towards AROPP. DARIPP does not induce a significant conformation in the L_β3–αC_ and L_β4–αD_ loops of AoRib2, whereas the L_αA–β1_ loop moves towards DARIPP. Therefore, AoRib2 possesses a different ligand-induced conformational change mechanism compared with BsRibG.

### 3.4. Conformational changes of AoRib2   

**Figure 5:**
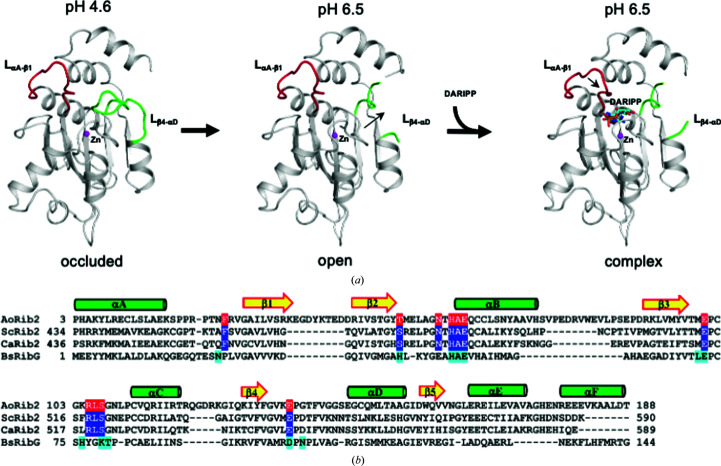
(*a*) Structural comparison of the occluded, open and complex structures. Structural differences were observed in the L_αA–β1_ (red) and L_β4–αD_ (green) loops. (*b*) Sequence alignment of the deaminase domains of AoRib2, ScRib2, *Candida albicans* Rib2 (CaRib2) and BsRibG. The substrate-binding residues in AoRib2 and BsRibG are shown in red and cyan, respectively; those predicted in ScRib2 and CaRib2 are shown in blue.

In the crystal structures of full-length AoRib2 and AoRib2_188_^pH4.6^, the L_β4–αD_ loop projects into the active site and sterically occludes the binding site for substrate entry. Due to the fact that the two crystal forms were crystallized under acidic conditions, AoRib2 tends to be in an occluded form at low pH. The structure of AoRib2_188_^pH4.6^ is virtually identical to that of AoRib2, with a root-mean-squared deviation (r.m.s.d.) of 0.25 Å for 186 C^α^ atoms. In AoRib2_188_^pH6.5^, the L_β4–αD_ loop becomes disordered (no electron density is visible for residues 139–143). The volume of the active site of the structure can accommodate its substrate. Therefore, the neutral pH structure can be considered as an open form. As DARIPP binds to AoRib2_188_, the L_αA–β1_ loop moves towards the active site in order to be sterically complementary to the substrate using van der Waals inter­actions and water-mediated hydrogen bonds. Most of the substrate-interacting residues do not change significantly, except Glu136, which rotates to interact with the O^2′^H group of the ribitol. The phosphate-binding site (L_β2–αB_ and L_β3–αC_ loops) adopts a similar conformation whether or not DARIPP is bound. This may indicate that the phosphate-binding site is structurally well organized in the absence of bound substrate. On the basis of these structures, we were able to distinguish three different conformations: an occluded form, an open form and a complex form [Fig. 5 ▸(*a*)]. These are likely to represent different functional states that would be controlled by the pH and the substrate.

Structural analysis of the occluded form reveals that the protonated O^ɛ1^ group of Glu100 forms a hydrogen bond to the backbone carbonyl group of Gly143 (2.7 Å). This interaction may contribute to the rigidity of the L_β4–αD_ loop. In the open form, a deprotonated Glu100 could not make close contacts with the L_β4–αD_ loop. Part of the L_β4–αD_ loop becomes disordered and allows substrate entry. Glu100 is highly conserved among fungal Rib2 deaminases [Fig. 5 ▸(*b*)], which leads to the possibility that the pH-triggered conformational switching mechanism could also apply to other Rib2s.

### 3.5. Importance of the C-terminal helix   

**Figure 6:**
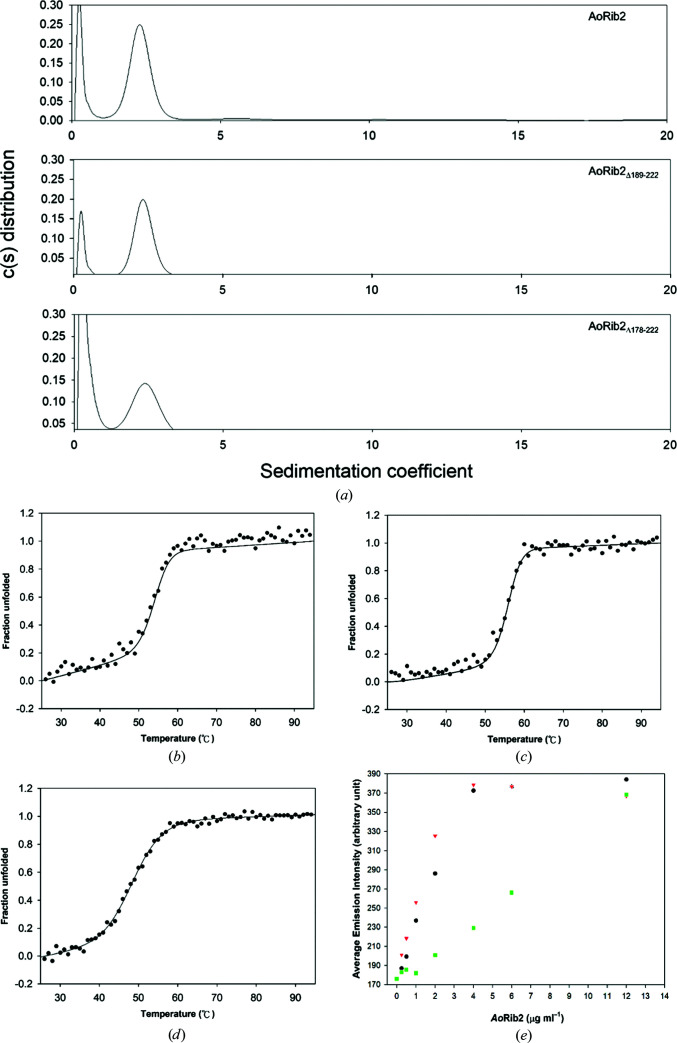
(*a*) Sedimentation-velocity analysis of AoRib2 deletion mutants. Top, AoRib2_188_; bottom, AoRib2_177_. The two deletion mutants were found to exist in a monomeric form. To compare protein stability, equilibrium thermal denaturation CD measurements of (*b*) AoRib2, (*c*) AoRib2_188_ and (*d*) AoRib2_177_ were conducted in 20 m*M* sodium phosphate pH 7.5. (*e*) A plot of the average fluorescence emission intensity against protein concentration for AoRib2 (black circles), AoRib2_188_ (red inverted triangles) and AoRib2_177_ (green squares).

Sequence analysis of fungal Rib2s shows that a stretch of additional residues are found at the C-terminus of AoRib2 that form a flexible C-terminal tail and helix αF. In order to understand the function of the flexible C-terminal tail and helix αF, two constructs with C-terminal deletions were generated (AoRib2_188_ and AoRib2_177_). The two deletion mutants were purified using the purification protocol used for AoRib2, but AoRib2_177_ had a slight tendency to aggregate. AoRib2_188_ and AoRib2_177_ were both found to be monomeric by analytical ultracentrifugation [Fig. 6 ▸(*a*)].

The *T*_m_ values of AoRib2 and the deletion mutants were measured by equilibrium thermal denaturation using circular-dichroism (CD) spectroscopy. AoRib2_188_ and the full-length protein have similar *T*_m_ values, but the *T*_m_ value of AoRib2_177_ is 5.4°C lower than that of the full-length protein [Figs. 6 ▸(*b*), 6 ▸(*c*) and 6 ▸(*d*)]. This can be explained by the structures: the flexible C-terminal tail is not involved in the structural core and does not influence the stability of the domain. The packing of helix αF against helix αE seems to increase the structural stability. In the deaminase assay, when the AoRib2 protein concentration increases the fluorescence intensity shows a two-state-like curve. The protein concentration at the midpoint of the transition is ∼2.5 µg ml^−1^ for the wild type and AoRib2_188_, while it is ∼6.6 µg ml^−1^ for AoRib2_177_ [Fig. 6 ▸(*e*)]. The AoRib2_177_ mutant lacking helix αF shows a reduced deaminase activity, which may be due to a decrease in the stability of the C-terminal structure. Helix αF plays an indispensable role in deaminase function. However, sequence analysis shows that the C-terminal helix αF of ScRib2 is shorter than that of AoRib2 [Fig. 5 ▸(*b*)]. This may indicate that ScRib2 has an alternative method of enhancing its structural stability. During evolution, nature has utilized distinct strategies to improve the structural stability of proteins, for instance through the formation of higher oligomers, more disulfide bonds, domain fusions, cofactor binding, salt bridges and hydrophobic patches. AoRib2 has longer loops and helix αF to increase the number of hydrophobic patches, whereas ScRib2 may use a domain-fusion strategy to compensate for the short C-terminal helix αF in structural stability.

## 4. Discussion   

Prior to this report, details of the three-dimensional structure of Rib2 and the residues involved in substrate binding were not known. Sequence and structural analysis demonstrated that Rib2 is a representative member of the CDA superfamily. The analysis suggests that the fungal Rib2 has high structural similarity to the eubacterial RibG. Therefore, these pyrimidine deaminases have a close evolutionary relationship. A structural comparison with RibG shows that they share a superimposable β-sheet and helices and that they adopt different conformations in the loops surrounding the active site. Despite their distinct substrate specificities for cyclic ribose versus linear ribitol, a portion of the residues are located in equivalent spatial positions. Similar to the BsRibG–AROPP complex, Glu63 O^ɛ1^ and Ala62 N form hydrogen bonds to the pyrimidine N^3^H and O^4^ groups, respectively, while Glu136 O^ɛ2^ interacts with the O^2′^H group of the ribitol. This AoRib2–DARIPP complex agrees with previous suggestions based on mutational analysis of ScRib2 (Chen *et al.*, 2013 ▸). Arg105 and Glu136 of AoRib2 directly interact with the ribitol and phosphate moieties of DARIPP, respectively. Mutation of the corresponding residues (Arg518 and Glu545) in ScRib2 significantly decreases the enzyme activity. This may indicate that Arg105 and Glu136 in AoRib2 are crucial for the specificity of its substrate. DARIPP binding could cause a slight conformational change in Rib2 and trigger changes in the protein dissimilar to those in RibG–AROPP. DARIPP triggers a conformational change in the L_αA–β1_ loop of AoRib2, while AROPP induces a change in the L_β3–αC_ and L_β4αD_ loops of RibG. This finding shows that the fungal Rib2 and the eubacterial RibG use different strategies to ensure substrate specificity.

**Figure 7:**
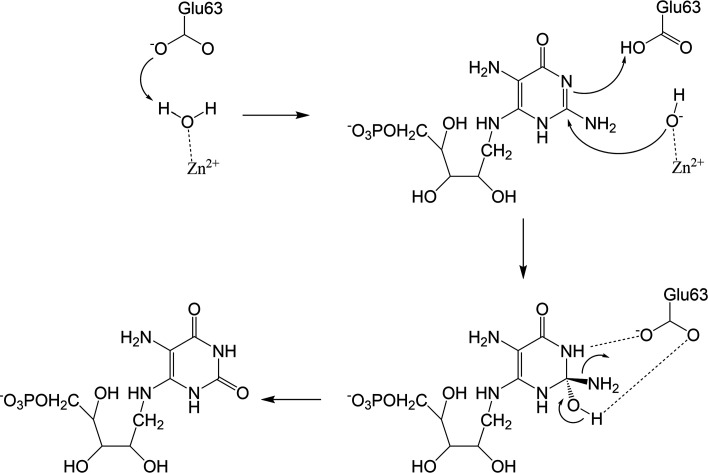
Proposed mechanism of deamination by AoRib2.

Previous studies of CDA superfamily members have demonstrated that they share a conserved hydrolytic deamin­ation mechanism (Liaw *et al.*, 2004 ▸; Betts *et al.*, 1994 ▸; Ko *et al.*, 2003 ▸; Kim *et al.*, 2006 ▸). Despite Rib2 and RibG acting on different substrates, they possess similar substrate-interacting networks with pyrimidine moieties and an identical catalytic center containing a zinc ion and a glutamate general base. It could be expected that Rib2 has a similar deamination mechanism to RibG (Chen *et al.*, 2013 ▸). Based on the AoRib2–DARIPP complex and previous studies of other CDA members, the deamination mechanism of Rib2 has been proposed (Fig. 7 ▸). The substrate DARIPP binds to the active site with its amino group stabilized by Glu63 O^ɛ2^ and Glu100 O. Glu63 is proposed to activate the nucleophile water molecule and facilitate attack on the C_2_ atom of the pyrimidine ring of DARIPP. The L_αA–β1_ loop moves towards DARIPP. Glu51 facilitates stabilization of the intermediate and proton transfer, producing ARIPP and an ammonium ion. Similar to the BsRibG–AROPP complex, the carbonyl group of ARIPP may bend out of the pyrimidine ring and ligate to the zinc ion. Glu63 O^ɛ2^ and Glu100 O create unfavorable contacts with the carbonyl group of ARIPP to promote product release.

To the best of our knowledge, most mononucleotide CDA members are oligomers. Yeast cytosine deaminase (yCD) and *B. subtilis* guanine deaminase (BsGD) are dimeric (Ko *et al.*, 2003 ▸; Liaw *et al.*, 2004 ▸). BsRibG, cytidine deaminase (CDA) and blasticidin-S deaminase (BSD) are tetrameric (Chen *et al.*, 2006 ▸; Sánchez-Quitian *et al.*, 2011 ▸; Kumasaka *et al.*, 2007 ▸). dCMP deaminase (dCMPD) is organized as hexamer (Hou *et al.*, 2008 ▸). Unexpectedly, AoRib2 is the only known monomeric mononucleotide deaminase in the CDA superfamily. Structural analysis of AoRib2 indicates that three loops (L_β1–β2_, L_αB–β3_ and L_αC–β4_) and longer secondary-structural elements (β1, β3 and αB) enlarge the structural core to maintain structural stability. These unique structural features may maintain a single deaminase domain of AoRib2 in solution. A structural comparison between AoRib2 and three members of the CDA superfamily shows the expected similarities in the three-layer α/β/α structure containing five β-strands (β1–β5) and three helices (αA–αC). A mutational analysis of AoRib2 shows that the C-terminal αF helix also contributes to structural stability and deaminase activity. Therefore, the diverse C-terminal tails in the CDA superfamily provide different functions.

Here, we present crystal structures of AoRib2 in open and occluded conformations. Structural comparisons of the conformations delineated the L_β4–αD_ loop involved in pH change-induced structural switching. In addition, we also determined the structure of AoRib2 in complex with DARIPP. A structural overlay of the open and complex structures revealed a slight conformational change induced by the substrate. The AoRib2–DARIPP complex explains how the fungal pyrimidine deaminase ensures its unique substrate specificity. AoRib2 shares structural similarity with the core of the deaminase domain of BsRibG, but shows divergence in the loops that provides its distinct substrate specificity. Additionally, AoRib2 possesses unique structural features, including a longer C-terminal helix αF, compared with ScRib2. The helix αF is crucial for maintaining structural stability and enzyme activity. These structural and functional studies of AoRib2 contribute to our further understanding of the biochemical functions of pyrimidine deaminases involved in riboflavin biosynthesis. Crystal structures of AoRib2 providing detailed stereochemistry of the active site and the binding of the DARIPP substrate should stimulate the design of antifungal inhibitors.

## Supplementary Material

PDB reference: Rib2, 7dry

PDB reference: Rib2 (1–188, pH 4.6), 7drz

PDB reference: Rib2 (1–188, pH 6.5), 7ds0

PDB reference: Rib2 (1–188, pH 6.5), complex with DARIPP, 7ds1

## Figures and Tables

**Table 1:** X-ray data-collection and refinement statistics Values in parentheses are for the highest resolution shell.

	Zn-SAD	AoRib2^pH4.6^	AoRib2_188_^pH4.6^	AoRib2_188_^pH6.5^	AoRib2_188_^pH6.5^–DARIPP
Data collection					
Wavelength (Å)	1.2824	0.9762	0.9762	1.0000	1.0000
Space group	*P*4_1_2_1_2	*P*4_1_2_1_2	*P*4_1_2_1_2	*P*2_1_2_1_2	*P*2_1_2_1_2
*a*, *b*, *c* (Å)	87.55, 87.55, 60.96	87.41, 87.41, 60.66	87.09, 87.09, 61.21	58.48, 90.90, 40.80	59.32, 90.77, 40.83
Resolution (Å)	30.00–2.20 (2.28–2.20)	30.00–1.44 (1.49–1.44)	30.00–1.70 (1.76–1.70)	30.00–1.70 (1.76–1.70)	30.00–1.58 (1.64–1.58)
Total observations	314250 (15847)	305782 (29940)	298500 (30924)	165158 (12677)	148862 (14668)
Unique reflections	12570 (1219)	43068 (4217)	26416 (2577)	25024 (2438)	31013 (3056)
Multiplicity	25.0 (13.0)	7.1 (7.1)	11.3 (12.0)	6.6 (5.2)	4.8 (4.8)
*R*_merge_ † (%)	12.6 (71.6)	4.2 (74.6)	10.1 (78.1)	8.9 (72.6)	4.5 (62.2)
*R*_p.i.m._ (%)	2.5 (19.4)	1.7 (30.0)	3.3 (23.6)	3.7 (37.0)	3.3 (34.8)
Completeness (%)	99.9 (99.8)	99.9 (100.0)	100.0 (100.0)	99.8 (99.5)	100.0 (100.0)
〈*I*/σ(*I*)〉	33.5 (3.5)	33.8 (2.2)	21.7 (3.1)	27.1 (2.4)	19.7 (2.1)
CC_1/2_	0.941 (0.591)	0.996 (0.805)	0.999 (0.887)	0.998 (0.757)	0.998 (0.919)
Refinement					
Resolution (Å)		30.00–1.44	30.00–1.70	30.00–1.70	30.00–1.58
No. of reflections (working/free)		41475/2097	26317/1292	23644/1139	30603/1538
*R*_work_ ‡ (%)		17.1 (21.4)	19.5 (22.4)	20.1 (20.7)	18.2 (22.5)
*R*_free_ ‡ (%)		19.7 (24.1)	21.0 (25.8)	21.6 (24.0)	20.7 (25.6)
R.m.s.d.					
Bond lengths (Å)		0.005	0.005	0.006	0.005
Bond angles (°)		0.84	0.78	0.69	0.69
Ramachandran plot (%)					
Most favored		98.9	98.9	98.3	98.9
Allowed		1.1	1.1	1.7	1.1
Average *B* values (Å^2^)					
Protein		20.8	18.9	19.7	19.8
Zinc ion		14.13	12.5	9.9	10.2
Sulfate ions		43.33	—	21.2	—
DARIPP		—	—	—	17.2
Water		36.72	28.7	29.0	29.8
PDB code		7dry	7drz	7ds0	7ds1

† *R*_merge_ = \textstyle \sum_{hkl}\sum_{i}|I_{i}(hkl)- \langle I(hkl)\rangle|/\textstyle \sum_{hkl}\sum_{i}I_{i}(hkl).

‡ *R*_work_ = \textstyle \sum_{hkl}\big ||F_{\rm obs}|-|F_{\rm calc}|\big |/ \textstyle \sum_{hkl}|F_{\rm obs}|. *R*_free_ was calculated using 5% of the data that were excluded from refinement.
